# Genetic susceptibility to cardiovascular disease and risk of dementia

**DOI:** 10.1038/tp.2017.110

**Published:** 2017-05-30

**Authors:** I K Karlsson, A Ploner, C Song, M Gatz, N L Pedersen, S Hägg

**Affiliations:** 1 Department of Medical Epidemiology and Biostatistics, Karolinska Institutet, Stockholm, Sweden; 2 Department of Psychology, University of Southern California, Los Angeles, CA, USA

## Abstract

Several studies have shown cardiovascular disease (CVD) to be associated with dementia, but it is not clear whether CVD *per se* increases the risk of dementia or whether the association is due to shared risk factors. We tested how a genetic risk score (GRS) for coronary artery disease (CAD) affects dementia risk after CVD in 13 231 Swedish twins. We also utilized summarized genome-wide association data to study genetic overlap between CAD and Alzheimer´s disease (AD), and additionally between shared risk factors and each disease. There was no direct effect of a CAD GRS on dementia (hazard ratio 0.99, 95% confidence interval (CI): 0.98–1.01). However, the GRS for CAD modified the association between CVD and dementia within 3 years of CVD diagnosis, ranging from a hazard ratio of 1.59 (95% CI: 1.05–2.41) in the first GRS quartile to 1.91 (95% CI: 1.28–2.86) in the fourth GRS quartile. Using summary statistics, we found no genetic overlap between CAD and AD. We did, however, find that both AD and CAD share a significant genetic overlap with lipids, but that the overlap arose from clearly distinct gene clusters. In conclusion, genetic susceptibility to CAD was found to modify the association between CVD and dementia, most likely through associations with shared risk factors.

## Introduction

Although cardiovascular disease (CVD) has an obvious role in vascular dementia (VaD), evidence indicates that vascular pathology is also of importance in Alzheimer's disease (AD).^1^ The exact nature of the association between CVD and dementia remains to be elucidated. Using data from the Swedish Twin Registry (STR),^2^ Eriksson *et al.*^3^ demonstrated a twofold increase in dementia incidence during the first 3 years following a diagnosis of non-stroke CVD. The authors also showed that the association was not a result of familial factors, but rather of the CVD *per se*. The two diseases share a number of risk factors such as hypertension, diabetes and dyslipidemia;^4^ it is not clear whether CVD is a causal factor increasing the risk of dementia, or whether the association can be explained by shared risk factors increasing the risk of both diseases.

Recent studies have indicated a decrease in dementia incidence,^5, 6, 7^ which may be attributable partly to better management of CVD and its risk factors. Hence, a better understanding of the underlying mechanisms linking CVD and its risk factors to dementia holds promise of preventive strategies and treatment regimens reducing the burden of both diseases.

Genome-wide association studies (GWAS) have identified 19 loci for AD^8^ and 55 for coronary artery disease (CAD).^9^ By using a genetic risk score (GRS) for CAD in a sample of 13 231 twins together with summary statistics from previously published GWAS, this study aims to understand the genetic association between dementia and CVD. More specifically, we aim to (i) investigate whether genetic susceptibility to CAD increases the risk of dementia and its subtypes, and (ii) investigate whether genetic susceptibility to CAD modifies the influence of prior non-stroke CVD diagnosis on dementia, (iii) explore the shared genetics of AD and CAD, as well as with their shared risk factors using summarized statistics.

## Materials and methods

### Study population

The STR is a nationwide registry containing twins born in Sweden between 1886 and 2008.^2^ Five substudies within the STR were used: The Swedish Adoption/Twin Study of Aging (SATSA),^10^ Origins of Variance in the Oldest Old: Octogenarian Twins (OCTO-Twin),^11^ Aging in Women and Men (GENDER),^12^ The Study of Dementia in Swedish Twins (HARMONY)^13^ and TwinGene.^2^ Briefly, SATSA (*n*=859) is a longitudinal study including physical and cognitive examinations on a 3-year rolling schedule across 30 years of time. OCTO-Twin is a longitudinal study of 351 same-sex twin pairs who were at least age 80 at baseline, and participated in up to five examinations every 2 years. GENDER is a longitudinal study of 249 unlike-sex twin pairs, consisting of three examinations every 4 years. Participants who at any wave showed indications of cognitive dysfunction were referred for a dementia evaluation. HARMONY is a cross-sectional study that started with a telephone screening for cognitive dysfunction of all twins in the STR aged 65 or older. All individuals who screened positive for cognitive dysfunction, their co-twin, and a control sample, were invited to participate in a clinical phase with physical and cognitive examination (*n*=1557). TwinGene is a cross-sectional study of 12 630 twins born before 1958 in Sweden. All individuals answered a mailed questionnaire and underwent health checkup. In total, genotype information is available for 13 231 individuals.

All participants provided informed consent and this study was approved by the Regional Ethics Board at Karolinska Institutet, Stockholm.

### Assessment of dementia

Dementia information was extracted from the National Patient Register (NPR), the Cause of Death Register (CDR), and the Prescribed Drug Register, the latter for AD only. All registries are nationwide and linked to the STR through the national personal identification number. Diseases are classified according to International Classification of Diseases (ICD) codes, and medications according to Anatomical Therapeutic Chemical codes. The sensitivity of register diagnoses for prevalent dementia is 63% when NPR and CDR are combined, and the specificity is over 98%.^14^ In addition, dementia was ascertained clinically in SATSA, OCTO-Twin, GENDER and HARMONY (*n*=4817 individuals), using a similar protocol described in detail elsewhere.^13^ To investigate whether the effect differed between dementia subtypes, dementia was further subdivided into AD and VaD. More information on registries, codes used and clinical work-up is reported in the Supplementary Document.

### Assessment of CVD

To gain power, we used all non-stroke CVD as exposure in analyses of the association between CVD and dementia. Diagnoses included are atherosclerosis, unstable angina, claudication, myocardial infarction and the surgical procedures coronary artery bypass grafting and percutaneous transluminal coronary angioplasty (Supplementary Table 1). A stricter definition, using only CAD as defined in the CARDIoGRAMplusC4D consortium,^9^ was used in sensitivity analyses. With this definition, only primary diagnoses of myocardial infarction or unstable angina were included. The validity of this definition has been shown to be at least 95% in the NPR.^15, 16^ Information about non-stroke CVD and CAD was gathered from the NPR.

### Genetic risk score

The CARDIoGRAMplusC4D consortium recently identified a total of 55 additive single-nucleotide polymorphisms (SNPs) robustly associated with CAD using 1000 genomes imputed data from 60 801 cases and 123 504 controls.^9^ Using these SNPs, we created an unweighted GRS for genetic susceptibility to CAD by summing the number of risk alleles. The GRS was used both as a continuous variable and categorized into quartiles. More information on genotyping and the GRS is in the Supplementary Section.

### Covariates

Covariates included in the primary analyses were age, sex, level of education and diagnosis of type 2 diabetes. Additional models included Apolipoprotein E (*APOE*) genotype, categorized into ε3/ε3 (*n*=7456), ε2 carriers (ε2/ε2 and ε2/ε3, *n*=1667) and ε4 carriers (ε3/ε4 and ε4/ε4, *n*=3617). *APOE* genotype was missing for 73 individuals. To compare the effect of the two alleles, those with the ε2/ε4 genotype (*n*=418) were excluded from these models. Sensitivity analyses were performed including prior stroke as a covariate. Education was dichotomized into less/more than 7 years of education, indicating basic versus higher education in Sweden at the time (information on education was missing for seven individuals). Information about prior stroke and diabetes was identified through the NPR (ICD-codes reported in Supplementary Table 1).

### Summarized data

We utilized publicly available summary statistics from GWASs of CAD^9^ and AD^8^ to investigate genetic overlap between the two diseases.

The data on CAD were contributed by the CARDIoGRAMplusC4D consortium, as mentioned above.

International Genomics of Alzheimer's Project (IGAP) is a large two-stage study based upon GWAS on individuals of European ancestry.^8^ We utilized data from stage 1, where IGAP used genotyped and imputed data on 7 055 881 SNPs to meta-analyze four previously published GWAS data sets consisting of 17 008 AD cases and 37 154 controls (The European Alzheimer's disease Initiative—EADI, the Alzheimer Disease Genetics Consortium—ADGC, The Cohorts for Heart and Aging Research in Genomic Epidemiology consortium—CHARGE and The Genetic and Environmental Risk in AD consortium—GERAD).

In addition, we used GWAS data for the shared risk factors blood pressure,^17^ blood lipids,^18^ body mass index (BMI)^19^ and type 2 diabetes^20^ to investigate their genetic overlap with AD and CAD.

### Statistical analyses

A Cox proportional hazard model with age as the underlying timescale was used to test the effect of the GRS and CVD on dementia and its subtypes. The models were stratified on study to allow for variation in the underlying hazard function across the included studies. CVD was treated as a time-dependent exposure; individuals were unexposed until the time of CVD diagnosis followed by two exposure levels; one during the first 3 years, and the second after more than 3 years post CVD diagnosis (as in Eriksson *et al.*^3^). To evaluate differences in the effect as a function of genetic risk, quartiles of the GRS were added as exposure to the model. To assess the linearity of the effect, another model included an interaction between CVD and quartiles of the GRS considered as a continuous measure. To evaluate differences in effect of the GRS on dementia by age, sex or *APOE* genotype an interaction term with these covariates was introduced. All above-mentioned analyses were performed using STATA 13.

To investigate genetic overlap between AD and CAD, the ‘VErsatile Gene-based Association Study’ (VEGAS) approach^21^ was used to calculate gene-level *P*-values from GWAS summary statistics. The method provides a measure of significance on the gene level by combining the signals from measured SNPs within the gene while accounting for underlying correlation patterns. Genes with a *P*-value below 2.84 × 10^−6^ were considered significant after Bonferroni correction for the 17 581 genes included. Overlapping genes were defined as having a *P*-value below the significance threshold for more than one of the phenotypes. *P*-values were calculated comparing the number of observed overlapping genes between two traits to what is expected under the null hypothesis using an exact binomial test. A heat map of genes associated with either AD or CAD and at least one of the shared risk factors was created showing the significance of genes across phenotypes. Lists of genes with a significant overlap between two traits were created, and used to identify pathways of importance for more than one outcome with the Consensus Path Database.^22^ This is a web-based tool for statistical testing of enrichment of selected genes in biological pathways with suitable adjustment for multiplicity (false discovery rate corrected *P*-values presented as q-values). The default settings for overrepresentation gene set analysis were used. More information on the gene-based analyses can be found in the supplement.

## Results

### Population characteristics

In total, 13 231 individuals were followed from 1 January 1978 or the age of 50 through 30 December 2014 or death, yielding 304 949 person years. During this period, 2630 events of CVD were identified. Mean age at CVD diagnosis was 70.0 years. The GRS for CAD significantly predicted CVD in the study population after adjusting for age, sex, education and diabetes (hazard ratio: 1.55, 95% confidence interval: 1.38–1.74 in the highest compared with the lowest quartile of the GRS). A total of 1430 individuals were diagnosed with dementia during follow-up. Of those, 868 were diagnosed with AD and 312 with VaD. Dementia cases were more likely to be female, have lower education, have a lower CAD GRS, and to have suffered from stroke and diabetes (Table 1). Mean age at dementia onset was 79.8 years.

### Association between the GRS and dementia

Using the Cox proportional hazard regression model, no association was found between the CAD GRS and dementia or its subtypes after adjusting for age, sex, education and diabetes. Nor was there any difference in dementia rate when the GRS was categorized into quartiles (Table 2). We found no evidence of an interaction between the GRS and age (*P*=0.66), sex (*P*=0.94) or *APOE* genotype (*P*=0.89).

### Association between CVD and dementia

We found a twofold increase in dementia hazard rate during the first 3 years following a CVD diagnosis (Table 3). After 3 years, however, the hazard rate was reduced to normal. A significant effect was seen both in AD and VaD, but the effect was much stronger in VaD.

### Association between CVD and dementia, stratified on GRS

When looking at the association between CVD and dementia stratified on quartiles of the CAD genetic score, we found the GRS to modify the association for dementia during the first 3 years following a CVD diagnosis (Table 3). The hazard ratio ranged from 1.59 in the lowest quartile of the GRS to 1.91 in the highest quartile, with a significant trend. No trend was seen for the effect of the GRS on the association between CVD and dementia more than 3 years after CVD diagnosis.

The modifying effect was seen for both AD and VaD within 3 years of CVD diagnosis stratified on quartiles of the GRS, but the magnitude of the estimates was higher for VaD. The trend remained significant for VaD, but not for AD, more than 3 years after CVD diagnosis.

Additional sensitivity analyses were performed by (i) censoring individuals after a stroke, (ii) only using clinical dementia diagnoses from the SATSA, OCTO-Twin, GENDER and HARMONY studies, (iii) using a strict definition of CAD as defined in the CAD GWAS,^9^ (iv) using a strict definition of the GRS excluding rs2075650 on the basis of proximity to the *APOE* gene and (v) adjusting for *APOE* genotype. The results did not change the interpretation of the main findings (Supplementary Table 2).

### Genetic overlap between AD and CAD

**Figure 1:**
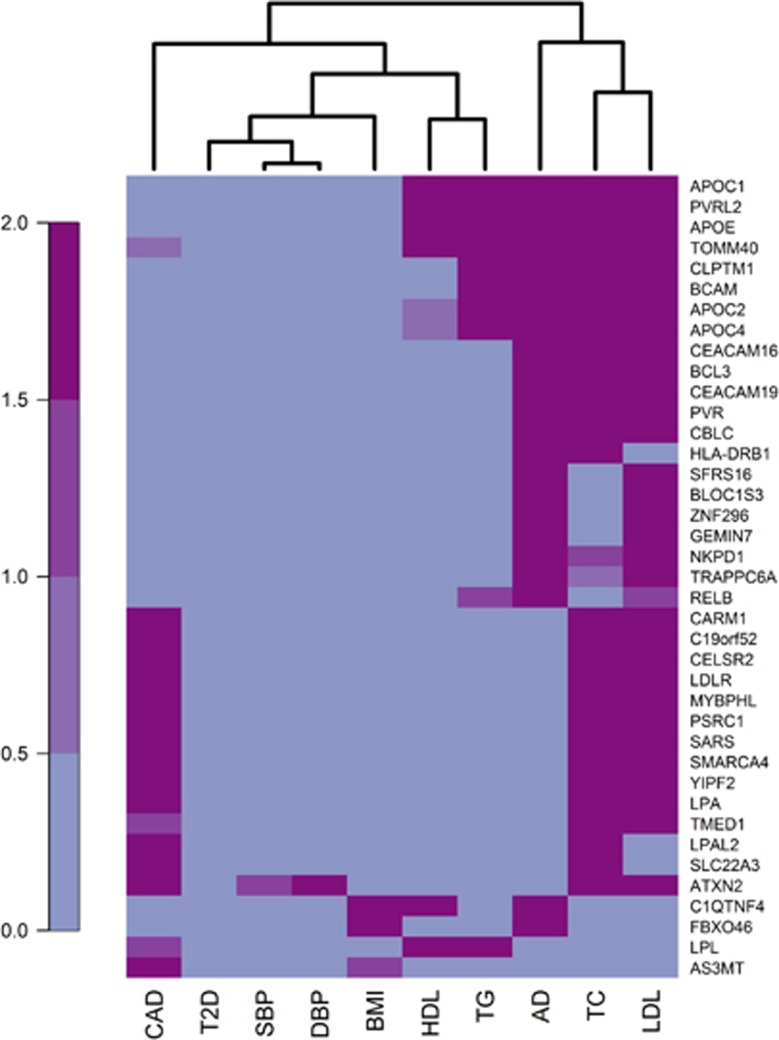
Heat map of significance of genes associated with AD or CAD across phenotypes. Each row represents one gene, and each column one phenotype. A total of 17 581 genes were available for all outcomes. Out of these, 39 genes were of significance for AD or CAD and at least one additional phenotype at the 2.84 × 10^−6^ level, and are hence included in the heat map. The *P*-values have been Bonferroni corrected for multiple testing and −log10 transformed. Blue color indicates no/low significance while purple indicates high significance. AD, Alzheimer's disease; BMI, body mass index; CAD, coronary artery disease; DBP, diastolic blood pressure; HDL, high-density lipoprotein; LDL, low-density lipoprotein; SBP, systolic blood pressure; T2D, type 2 diabetes.

The heat map of significance of the genes associated with AD or CAD across phenotypes showed no common cluster of significant genes for AD and CAD (Figure 1). Both AD and CAD shared gene clusters with lipid fractions, but the clusters were clearly separate from each other. The gene-based tests demonstrated that AD and CAD each had a significant number of genes in common with low-density lipoprotein cholesterol and total cholesterol, but not with the same genes, whereas only AD overlapped with high-density lipoprotein cholesterol and triglycerides (Table 4). AD also had a significant number of genes in common with BMI, while CAD had genes in common with systolic blood pressure. Neither AD nor CAD seemed to have any genetic overlap with type 2 diabetes (Figure 1 and Table 4). The lists of overlapping genes of significance are provided in Supplementary Table 3.

A closer look at intersecting gene clusters using pathway analyses identified 17 pathways for genes in common to lipids and AD, and 13 pathways for genes in common to lipids and CAD (Supplementary Table 4). Out of these, six pathways were identified both for lipids and AD and lipids and CAD: the statin pathway (*q*=5.82 × 10^−5^ and *q*=9.88 × 10^−4^), chylomicron-mediated lipid transport (*q*=7.95 × 10^−4^ and *q*=4.36 × 10^−4^), lipoprotein metabolism (*q*=1.40x10^−3^ and *q*=3.70 × 10^−5^), retinoid metabolism and transport (*q*=2.48 × 10^−3^ and *q*=1.50 × 10^−3^), lipid digestion, mobilization and transport (*q*=3.88 × 10^−3^ and *q*=9.26 × 10^−5^) and visual phototransduction (*q*=7.95 × 10^−3^ and *q*=4.70 × 10^−3^).

## Discussion

In this large study of 13 231 Swedish twins, we found an increased dementia risk during the first 3 years after a CVD diagnosis. A GRS for CAD modified the association between CVD and dementia, suggesting that genetically predisposed CVD is a stronger risk factor for dementia than CVD with a lower genetic risk. However, the GRS for CAD did not increase dementia risk directly. Utilizing a gene-based approach with summary statistics from previously published GWAS, we found no genetic overlap between CAD and AD. We did, however, find a significant excess in genes in common with lipid fractions for both CAD and AD. These results indicate that the association between CVD and dementia is not due to genetic overlap, rather, the association may be a consequence of lipid dysregulation increasing the risk of both diseases.

The temporal effect of CVD on dementia has been shown previously, both in a related sample of Swedish twins and in the Rotterdam study.^3, 23^ Considering the lack of genetic overlap and the long preclinical phase of dementia, this may indicate that the CVD event acts as a biological or environmental stressor further promoting dementia development. As dementia pathology starts long before diagnosis, it is plausible that the time-dependent effect of CVD lies in bringing susceptible individuals over a threshold while not affecting more resilient individuals. Our finding of increased risk as a function of genetic risk for CAD may provide clues to which individuals are more or less resilient to these stressors.

CVD and dementia are both heterogeneous diseases, caused by a multitude of genetic and environmental factors as well as interactions between them. The manner in which genetic susceptibility to CAD modified the association between CVD and dementia suggests that genetically predisposed CVD is a stronger risk factor for dementia than CVD with a low genetic risk. However, several of the SNPs in the GRS are also associated with risk factors for both CVD and dementia. Seven of the top SNPs identified for CAD reside in genes of importance for lipid levels, and four in genes important for blood pressure.^17, 18^ Hence, it is plausible that the modifying effect of the CAD GRS on the association between CVD and dementia stems from genetic susceptibility to lipid dysregulation rather than to CVD itself.

The gene-based analyses highlighted a clear distinction between the lipid-related genes in common with CAD from those in common with AD. Pathway analyses further identified lipid metabolism and related pathways as the most important pathways, and that the relevant genes were different for CAD and AD. Cholesterol is one of the most well-established risk factors for CVD.^24^ It is also highly relevant to dementia, with the brain harboring approximately 25% of the body’s cholesterol.^25^ Statins are commonly used in the primary and secondary prevention of CVD and affect disease pathology mainly through lowering circulating cholesterol levels. Several observational studies have shown statins to significantly lower the risk of AD, although randomized controlled trials found no such benefit.^26^ As the brain has its own cholesterol metabolism separated from that in the periphery by the blood–brain barrier, it is plausible that the same biological pathways influence the risk of both CVD and dementia, but through different risk genes. *APOE*, by far the most important genetic risk factor for AD, is the main cholesterol transporter in the brain.^27^ The ε4 risk allele increases the risk of both dementia and CVD while the ε2 allele increases the risk of CVD while having protective effect on dementia.^25^ Using a subset of the study sample included here, Eriksson *et al.*^3^ showed that only carriers of the ε4 allele had an increase in dementia rate after suffering from a non-stroke CVD. However, including *APOE* as a covariate in the models did not affect our findings and there was no evidence of an interaction between *APOE* and the GRS. The gene-based results identified *APOE* as significant for AD as well as all four lipid fractions, but not for CAD. Taken together, this is in line with previous evidence of the effect of *APOE* on CVD operating mainly through lipid dysregulation, while the effect on AD operates also through other mechanisms.^28^

Using LD score regression, Bulik-Sullivan *et al.*^29^ investigated genome-wide correlations across multiple traits, but did not find evidence of a correlation between AD and CAD. As visualized in Supplementary Figure 1, all shared risk factors covered in our study had genetic correlations with CAD, but only high-density lipoprotein cholesterol had a very weak positive genetic correlation with AD. In contrast, our gene-based results showed clusters of genes shared by AD and all four lipid fractions and BMI. Furthermore, although LD score regression demonstrated significant genetic correlations between CAD and type 2 diabetes and triglycerides,^29^ our gene-based analyses did not find a significant number of genes shared by these phenotypes. Although the use of different sets of summarized data for CAD might explain the differences in the results to some extent, it is unlikely to be the full explanation. One possible reason for the discrepancy is that the methods operate on different genetic levels. LD score regression uses all available SNPs across the entire genome, while the gene-based method focuses on functional genes and will hence miss signals in noncoding regions. Furthermore, while the VEGAS approach uses the *P*-value for each SNP within a gene to assign a gene-based significance, the LD score regression method uses the β-value of each SNP and hence also considers the direction of the effect. It is therefore plausible that several small effects with opposite direction cancel out in LD score regression due to the underlying genetic architecture. On the other hand, the VEGAS approach allows for allelic heterogeneity, arising from multiple variants within the same gene affecting the same phenotype, and might catch additional effects missed by the LD score regression method.

If the association between CVD and dementia in fact suggested a causal effect of CVD on dementia, some evidence of an association between the CAD GRS and dementia should be evident. One method for assessing causality is using Mendelian randomization techniques,^30^ where SNPs related to the exposure are used as an instrumental variable to assess a causal effect on the outcome. One of the core assumptions of Mendelian randomization is that there is no pleiotropy, that is, other biological pathways than through the exposure, between the SNPs used as instrumental variable and the outcome. In the case of CAD and AD, we present evidence of lipid-related genes associated with both CAD and AD, providing another possible path from SNP to outcome, hence violating the pleiotropy assumption. Therefore, a full Mendelian randomization study on CVD and dementia would not be valid to answer the question of causality.

The results presented here are based on a large cohort of twins with up to 37 years of follow-up. The use of summary statistics from large GWAS studies further strengthens the study. By using gene-based analysis and comparing the results with those from Bulik-Sullivan *et al.*,^29^ in most cases using the same data, we could further highlight the complex genetic architecture of CAD and AD. The linkage to nationwide registers further strengthens the study, but also carries some limitations. Disease diagnoses from registers for conditions with different degrees of severity may induce a misclassification bias, where individuals suffering from more severe disease are more likely to be identified. Considering the near-perfect specificity of the NPR and CDR,^14^ the low sensitivity would not influence the results as long as any misclassification is non-differential. As information about both exposure and outcome was gathered from the registers, there is a risk that diagnosis of one disease makes a register record of the other more likely, hence inflating the results. However, if such a bias existed, it would be evident when comparing the main results to the sensitivity analysis using only clinically assessed dementia, which showed similar results for the effect of CVD on dementia. Similarly, sensitivity analyses using the validated definition of CAD showed similar estimates as the main analyses. As both CVD and dementia are diseases with a long preclinical phase, it can be difficult to determine disease onset, which may very well be several years before diagnosis. The strict definition of CAD uses only primary diagnoses of the more severe forms of disease, and should hence be able to estimate onset more precisely. Similarly, the clinically assessed dementia used both informant interviews and review of medical records to better estimate age at onset. Another issue is the distinction between AD and VaD using register data. Although results in sensitivity analyses of clinically assessed AD and VaD showed similar results, the sample size for the stratified analyses was small and interpretation of the differences in dementia subtypes should therefore be made with caution.

One problem worth mentioning in studies of risk factors for dementia is taking the temporal association into account. For example, high BMI increases the risk of dementia when measured in midlife,^31^ but is associated with a decrease in risk when measured in late life.^32^ The same pattern has been shown for high blood pressure, with studies showing an increased dementia risk when measured in midlife and discrepant results for late life.^33^ Although genotypes are stable throughout life, their effect on dementia risk may not be. Although we found no evidence of effect modification by age, this factor may further complicate studies of dementia and genetic correlations and warrants further investigation.

In conclusion, we found genetically predisposed CVD to be a stronger risk factor for dementia than CVD with a lower genetic risk. The effect cannot be explained by a genetic overlap between the diseases, but may be due to shared influences via lipid metabolism.

## Figures and Tables

**Table 1:** Demographic characteristics of dementia cases and controls

	*No dementia* *(*n=*11 975)*	*All dementia* *(*n=*1256)*	*Alzheimer'*s*disease* *(*n=*741)*	*Vascular demented* *(*n=*273)*
Age at baseline, mean (s.d.)	50.7 (2.8)	54.7 (5.7)^a^	54.8 (5.8)^a^	54.6 (5.6)^a^
Age at death, mean (s.d.)	81.5 (9.2)	86.0 (6.2)^a^	86.5 (6.2)^a^	85.5 (5.8)^a^
Follow up time, mean (s.d.)	22.8 (8.3)	25.2 (7.0)^a^	25.1 (6.9)^a^	25.0 (6.4)^a^
GRS for CAD, mean (s.d.)	53.9 (4.4)	53.5 (4.3)^a^	53.6 (4.4)^a^	53.4 (4.2)
Female sex, *n* (%)	6206 (52.6)	840 (59.7)^a^	548 (63.1)^a^	149 (47.8)
Low education, *n* (%)	3877 (33.0)	840 (58.9)^a^	492 (56.9)^a^	187 (60.1)^a^
Non-stroke CVD, *n* (%)	2324 (19.7)	306 (21.4)	148 (17.1)	90 (28.9)^a^
Prior stroke, *n* (%)^b^	1057 (9.0)	241 (16.9)^a^	65 (7.5)	122 (39.1)^a^
Diabetes, *n* (%)^b^	1145 (9.7)	167 (11.7)^a^	81 (9.3)	46 (14.7)^a^

Abbreviations: CAD, coronary artery disease; CVD, cardiovascular disease; GRS, genetic risk score.

a Significant difference compared with the no dementia group (*P*<0.05) based on chi-square test for binary variables and *t*-test for continuous variables.

b Stroke and diabetes before dementia onset.

Number of exposed individuals (percent of number of individuals with covariate data) for binary variables, and mean age (standard deviation) for continuous variables.

**Table 2:** Hazard ratios of dementia in relation to genetic risk of coronary artery disease

	*All dementia*	*Alzheimer's disease*	*Vascular dementia*
GRS for CAD	1.01 (1.00–1.02)	1.01 (0.99–1.03)	1.01 (0.98–1.04)
1st quartile	1.00	1.00	1.00
2nd quartile	0.99 (0.85–1.14)	0.96 (0.78–1.18)	0.98 (0.71–1.36)
3rd quartile	1.05 (0.90–1.22)	0.96 (0.78–1.19)	1.18 (0.86–1.62)
4th quartile	1.10 (0.95–1.29)	1.20 (0.98–1.48)	0.85 (0.59–1.24)

Abbreviations: CAD, coronary artery disease; GRS, genetic risk score.

Hazard ratios (95% confidence intervals) of dementia/Alzheimer's disease in relation to genetic risk of CAD, both as continuous measure and categorized into quartiles. The model is adjusted for age, sex, education and diabetes before dementia onset.

**Table 3:** Hazard ratios of dementia during the first three years following CVD diagnosis, and more than three years following a CVD diagnosis, for total sample and stratified on genetic risk score for coronary artery disease

	*Total sample*	*Stratified on CAD genetic risk score*				
		*1st quartile*	*2nd quartile*	*3rd quartile*	*4th quartile*	*Trend* P*-value*
*All dementia cases*	n=*1430*	n=*392*	n=*356*	n=*364*	n=*318*	
HR first 3 years after CVD	**1.92 (1.57–2.36)**	**1.59 (1.05–2.41)**	**1.82 (1.19–2.78)**	**2.38 (1.65–3.42)**	**1.91 (1.28–2.86)**	**<0.000001**
HR>3 years after CVD	1.08 (0.92–1.26)	1.11 (0.81–1.51)	0.84 (0.61–1.18)	**1.34 (1.02–1.78)**	1.02 (0.74–1.40)	0.35
						
*Alzheimer's disease cases*	n=*868*	n=*235*	n=*209*	n=*211*	n=*213*	
HR first 3 years after CVD	**1.47 (1.11–1.95)**	1.65 (0.97–2.79)	1.36 (0.75–2.48)	1.09 (0.56–2.12)	1.64 (1.00–2.70)	**0.02**
HR>3 years after CVD	0.84 (0.67–1.05)	0.96 (0.63–1.49)	0.81 (0.52–1.25)	0.88 (0.58–1.35)	0.71 (0.47–1.08)	0.7
						
*Vascular dementia cases*	n=*312*	n=*85*	n=*83*	n=*86*	n=*58*	
HR first 3 years after CVD	**2.68 (1.85–3.89)**	1.30 (0.52–3.30)	1.80 (0.77–4.18)	**5.61 (3.24–9.71)**	**2.56 (1.08–6.08)**	**<0.000001**
HR>3 years after CVD	1.35 (0.99–1.83)	0.76 (0.37–1.57)	1.04 (0.57–1.89)	**1.99 (1.15–3.42)**	**1.92 (1.05–3.48)**	**0.01**

Abbreviations: CAD, coronary artery disease; CVD, cardiovascular disease; HR, hazard ratio.

Number of cases and hazard ratios (95% confidence intervals) of dementia/Alzheimer's disease in the presence of CVD, for the total sample and stratified by quartiles of genetic risk score for CAD. Bold numbers indicate significance. The model is adjusted for age, sex, education and diabetes during follow-up.

**Table 4:** Pairwise genetic overlap between Alzheimer's disease, coronary artery disease and their shared risk factors

	*Nr of genes*	*Alzheimer's disease*			*Coronary artery disease*		
		*Expected*	*Observed*	P*-value*	*Expected*	*Observed*	P*-value*
Alzheimer's disease	42	—	—	—	0	0	1
Coronary artery disease	54	0	0	1	—	—	—
Type 2 diabetes	11	0	0	1	0	0	1
Body mass index	56	0	2	**0.04**	0	0	1
Total cholesterol	231	1	14	**1.03 × 10**^−**15**^	1	14	**3.02 × 10**^−**14**^
High-density lipoprotein	198	0	5	**1.33 × 10**^−**4**^	1	1	0.46
Low-density lipoprotein	151	0	20	**3.52 × 10**^**−28**^	0	12	**1.24 × 10**^**−13**^
Triglycerides	175	0	8	**1.28 × 10**^**−8**^	1	1	0.41
Systolic blood pressure	15	0	0	1	0	1	**0.05**
Diastolic blood pressure	24	0	0	1	0	1	0.07

Table of the total number (Nr) of significant genes for each phenotype at *P*<2.84 × 10^**−**6^ out of the 17 581 genes available. For Alzheimer's disease and coronary artery disease, the expected and observed number of genes in common with risk factors, together with Fisher *P*-values for the observed overlap. Bold numbers indicate significance.
