# Supplementary material for: Genetic susceptibility to cardiovascular disease and risk of dementia
Source: Transl Psychiatry. 2017 May 30;7(5):e1142–. doi: 10.1038/tp.2017.110 (PMC5534941; doi:10.1038/tp.2017.110)
Supplement: Supplementary Information [file tp2017110x1.docx]

**Supplementary section**

**National Disease Registers**

The Swedish Twin Registry (STR) is linked to several population-based registers by the Swedish 10-digit personal identification number. Used here are the National Patient Registry (NPR), the Cause of Death Registry (CDR), and the Prescribed Drug Registry (PDR).

The NPR contains information about all in-patient care at hospitals in Sweden. The registry was initiated 1964 and reached nationwide coverage 1987. It now includes 99% of all overnight hospitalizations^1^. Each record corresponds to one hospitalization, with the primary diagnosis together with up to eight additional diagnoses. Since 2001, the register also covers outpatient specialist care in hospitals. Diseases are classified according to International Classification of Diseases (ICD) codes, and dementia could hence be subdivided into Alzheimer´s disease (AD) and vascular dementia (VaD) (Table S1). ICD-7 was used prior to 1969, ICD-8 between 1969 and 1986, ICD-9 from 1987 until 1996, and ICD-10 from 1997 and onwards.

The CDR reached nationwide coverage in 1961 and includes information about the underlying and contributory causes of death, also given in ICD codes. This register includes all individuals, who in the year of death were registered as residents in Sweden. Data from NPR and CDR were available until the end of 2014. For all phenotypes included in this study, both primary and additional diagnoses are used as criteria for disease.

The PDR contains information on all prescriptions dispensed classified according to Anatomical Therapeutic Chemical (ATC) codes. The register reached nation-wide coverage in 2005, and is currently updated until the end of 2015. We used information on medication use from 2005 and throughout 2014. All dementia medication currently used in Sweden is specific to AD, and the register was hence used as a source to identify AD cases. Medications prescribed in Sweden in the ATC group N06D were used to identify individuals prescribed AD medications (N06DA02-04 and N06DX01).

**Clinical dementia ascertainment**

Dementia was ascertained clinically in the SATSA, OTCTO-Twin, GENDER, and HARMONY studies^2^. In brief, a cognitive screening was performed using the Mini-Mental State Examination ^3^, alternatively by TELE for telephone screening with individuals and informants^4^. Screening was followed by a clinical work-up of suspected cases and their co-twins, including cognitive testing, physical and neurological work-ups, informant interviews, reviews of medical records and laboratory tests. Final dementia diagnosis was set at multidisciplinary consensus conferences, according to DSM-III-R^5^ or DSM-IV^6^ criteria. Dementia was further differentially diagnosed into AD according to the NINCDS/ADRDA criteria^7^, and VaD according to the NINDS/AIREN criteria^8^ .

**Genotyping and creation of the genetic risk score**

TwinGene participants were genotyped using Illumina OmniExpress and imputation was done based on the 1000 Genomes Project phase 1 ver 3 data^9^. SATSA, OCTO-Twin, GENDER, and HARMONY were genotyped using CardioMetabochip, a customized chip that prioritizes loci based on a previous genome-wide association studies (GWASs) for cardiovascular and metabolic traits^10^.

Apolipoprotein E (*APOE*) was directly genotyped in SATSA, OCTO-Twin, GENDER, and HARMONY, while it was imputed for TwinGene participants against the 1000 genomes panel^11^.

To create the genetic risk score (GRS), we utilized 55 single nucleotide polymorphisms (SNPs) robustly associated with coronary artery disease (CAD)^9^. Only additive SNPs were included. When more than one SNP was identified within the same locus, the SNP with the lowest p-value was selected. Values for missing SNPs were imputed based on the effect/non-effect allele frequency. 18 of the SNPs were not present on the CardioMetabochip. For 11 of these a proxy was used, and for the remaining 7 SNPs the value was imputed. A list of the SNPs and proxies used can be found in Supplementary Table 5.

The genetic risk score for CAD ranged between 36.88 and 70.66. Quartiles were created with cut-offs at 50.75, 53.73, and 56.78.

**Gene-based analyses**

To retrieve lists of gene-based p-values, we downloaded the summary statistics for each phenotype (AD^12^, CAD^13^, body mass index^14^, type 2 diabetes^15^, systolic- and diastolic blood pressure^16^, high density lipoporotein cholesterol, low density lipoprotein cholesterol, triglycerides, and total cholesterol^17^) and applied the ‘VErsatile Gene-based Association Study‘ (VEGAS) software, described in detail elsewhere^18^. In brief, the method sums the χ^2^-converted p-values of all SNPs within a gene into a gene-based test statistic, after accounting for LD by using simulations from HapMap LD structures. By doing so, statistical power can be increased and the burden of multiple testing reduced. The maximum number of simulations was set to 1 000 000 to balance computational burden and ability to compute p-values down to 10^-6^ (p-value threshold of significance of 2.84x10^-6^ after Bonferroni-correction). SNPs were assigned to genes using the genome browser hg18 assembly, rendering 17 581 genes available for all phenotypes.

In the next step, VEGAStools^19^, an R package developed for manipulation and display of gene lists produced by the VEGAS software, was used to compare overlap, create a heatmap, and produce gene lists.

The Consensus Path DB was used to carry out analysis of enrichment pathways. The method utilizes 12 databases to identify pathways enriched for the selected gene sets. Q-values are calculated from false discovery rate corrected p-values based on the hypergeometric formula. The method is described in detail elsewhere^20^.

**Supplementary Table 1: ICD-codes for identification of disease**

|  | **ICD-7** | **ICD-8** | **ICD-9** | **ICD-10** | **Surgical code** |
| --- | --- | --- | --- | --- | --- |
| **Dementia** |  |  |  |  |  |
| **Alzheimer´s disease** | 304 | 290 | 290A | F00 |  |
|  | 305 |  | 290B | G30 |  |
|  |  |  | 311A |  |  |
|  |  |  |  |  |  |
| **Vascular dementia** |  | 293.0 | 290E | F01 |  |
|  |  | 293.1 |  |  |  |
|  |  |  |  |  |  |
| **Other dementia** | 306 |  | 290X | F02 |  |
|  |  |  | 290W | F03 |  |
|  |  |  | 294B | G311 |  |
|  |  |  | 311B | G318A |  |
|  |  |  | 311C | F051 |  |
|  |  |  | 311X |  |  |
|  |  |  |  |  |  |
| **CVD** |  |  |  |  |  |
| **Non-stroke CVD** | 420 | 410 | 410 | I20 | 984 |
|  | 450 | 411 | 411 | I21 | 3068 |
|  | 453.33 | 412 | 412 | I22 | 3080 |
|  |  | 413 | 413 | I23 | 3127 |
|  |  | 414 | 414 | I24 | 3141 |
|  |  | 440 | 440 | I25 | 3158 |
|  |  | 443.90 | 443X | I79 | FNC |
|  |  |  |  | I73.9 | FND |
|  |  |  |  |  | FNE |
|  |  |  |  |  | FNG00 |
|  |  |  |  |  | FNG02 |
|  |  |  |  |  | FNG05 |
|  |  |  |  |  |  |
| **Strict definition of CAD** |  | 410 | 410 | I21 | 3080 |
|  |  | 411 | 411B | I22 | 3127 |
|  |  |  |  | I20.0 | 3158 |
|  |  |  |  |  | FNC |
|  |  |  |  |  | FND |
|  |  |  |  |  | FNE |
|  |  |  |  |  | FNG02 |
|  |  |  |  |  | FNG05 |
|  |  |  |  |  |  |
| **Stroke** | 330 | 430 | 430 | I60 |  |
|  | 331.00 | 431 | 431 | I61 |  |
|  | 331.01 | 433 | 434 | I63 |  |
|  | 331.09 | 434 | 436 | I64 |  |
|  | 331.99 | 436 |  |  |  |
|  | 332.00-19 |  |  |  |  |
|  | 332.29 |  |  |  |  |
|  | 334.00-98 |  |  |  |  |
|  |  |  |  |  |  |
| **Diabetes** | 260 | 250 | 250 | E10 |  |
|  |  |  |  | E11 |  |
|  |  |  |  | E12 |  |
|  |  |  |  | E13 |  |
|  |  |  |  | E14 |  |

Abbreviations: ICD, international classification of disease; CVD, cardiovascular disease; CAD, coronary artery disease

**Supplementary Table 2: Sensitivity analyses: Hazard ratios of dementia during the first three years following CVD diagnosis, and more than three years following a CVD diagnosis for total sample and stratified on genetic risk score for coronary artery disease.**

1. **Censoring individuals after a stroke**

|  | Total sample | | Stratified on CAD genetic risk score | | | | | | | | |
| --- | --- | --- | --- | --- | --- | --- | --- | --- | --- | --- | --- |
|  |  |  | **1^st^ quartile** | | **2^nd^ quartile** | | **3^rd^ quartile** | | **4^th^ quartile** | | **Trend p-value** |
| All dementia cases |  | n=1251 |  | n=348 |  | n=317 |  | n=304 |  | n=282 |  |
| HR first 3 y after CVD | **1.72** | **(1.37-2.17)** | **1.57** | **(1.00-2.48)** | 1.50 | (0.90-2.49) | **2.33** | **(1.54-3.53)** | 1.51 | (0.93-2.45) | **p<0.000001** |
| HR > 3 y after CVD | 1.00 | (0.83-1.19) | 1.07 | (0.75-1.51) | 0.85 | (0.59-1.22) | 1.15 | (0.82-1.61) | 0.93 | (0.65-1.33) | p=0.91 |
| Alzheimer´s disease cases |  | n=816 |  | n=226 |  | n=198 |  | n=193 |  | n=199 |  |
| HR first 3 y after CVD | **1.40** | **(1.04-1.88)** | **1.85** | **(1.11-3.08)** | 1.23 | (0.63-2.39) | 1.04 | (0.51-2.12) | 1.34 | (0.76-2.35) | p=0.12 |
| HR > 3 y after CVD | 0.86 | (0.69-1.08) | 0.99 | (0.63-1.54) | 0.87 | (0.56-1.36) | 0.83 | (0.53-1.31) | 0.74 | (0.48-1.14) | p=0.11 |
| Vascular dementia cases |  | n=227 |  | n=62 |  | n=62 |  | n=59 |  | n=44 |  |
| HR first 3 y after CVD | **2.04** | **(1.34-3.10)** | 1.40 | (0.56-3.51) | 1.25 | (0.45-3.46) | **3.68** | **(1.95-6.96)** | 2.05 | (0.83-5.04) | **p=0.0001** |
| HR > 3 y after CVD | 1.17 | (0.84-1.63) | 0.75 | (0.35-1.61) | 0.98 | (0.51-1.86) | 1.59 | (0.89-2.83) | 1.46 | (0.75-2.84) | p=0.13 |

**b) Using clinically assessed dementia.**

|  | Total sample | | Stratified on CAD genetic risk score | | | | | | | | |
| --- | --- | --- | --- | --- | --- | --- | --- | --- | --- | --- | --- |
|  |  |  | **1^st^ quartile** | | **2^nd^ quartile** | | **3^rd^ quartile** | | **4^th^ quartile** | | **Trend p-value** |
| All dementia cases |  | n=694 |  | n=214 |  | n=179 |  | n=166 |  | n=135 |  |
| HR first 3 y after CVD | **2.11** | **(1.58-2.83)** | **2.13** | **(1.27-3.57)** | 1.19 | (0.55-2.57) | **3.86** | **(2.39-6.22)** | 1.41 | (0.69-2.88) | **p=0.000003** |
| HR > 3 y after CVD | 1.21 | (0.92-1.58) | 1.08 | (0.65-1.81) | 1.18 | (0.72-1.93) | 1.55 | (0.93-2.57) | 1.07 | (0.58-1.97) | p=0.18 |
| Alzheimer´s disease cases |  | n=392 |  | n=117 |  | n=101 |  | n=89 |  | n=85 |  |
| HR first 3 y after CVD | **1.66** | **(1.08-2.56)** | **2.27** | **(1.14-4.49)** | 0.61 | (0.15-2.45) | **2.47** | **(1.08-5.65)** | 1.39 | (0.57-3.42) | p=0.05 |
| HR > 3 y after CVD | 0.94 | (0.62-1.44) | 0.89 | (0.39-2.04) | 1.11 | (0.55-2.26) | 1.01 | (0.41-2.49) | 0.71 | (0.28-1.82) | p=0.68 |
| Vascular dementia cases |  | n=179 |  | n=54 |  | n=48 |  | n=52 |  | n=25 |  |
| HR first 3 y after CVD | **3.40** | **(2.13-5.40)** | 2.39 | (0.92-6.22) | 1.64 | (0.50-5.35) | **8.88** | **(4.73-16.64)** | 0.83 | (0.11-6.24) | **p<0.000001** |
| HR > 3 y after CVD | 1.43 | (0.90-2.28) | 0.46 | (0.11-1.91) | 1.63 | (0.75-3.53) | 2.09 | (0.92-4.77) | 1.85 | (0.68-5.00) | **p=0.04** |

**c) Using a strict definition of CAD**

|  | Total sample | | Stratified on CAD genetic risk score | | | | | | | | |
| --- | --- | --- | --- | --- | --- | --- | --- | --- | --- | --- | --- |
|  |  |  | **1^st^ quartile** | | **2^nd^ quartile** | | **3^rd^ quartile** | | **4^th^ quartile** | | **Trend p-value** |
| All dementia cases |  | n=1430 |  | n=392 |  | n=356 |  | n=364 |  | n=318 |  |
| HR first 3 y after CVD | **2.16** | **(1.68-2.78)** | **1.82** | **(1.06-3.11)** | **2.04** | **(1.21-3.44)** | **3.26** | **(2.11-5.02)** | 1.62 | (0.93-2.83) | **p<0.000001** |
| HR > 3 y after CVD | 1.09 | (0.89-1.33) | 1.16 | (0.78-1.72) | 0.91 | (0.60-1.38) | 1.41 | (1.00-1.98) | 0.88 | (0.57-1.35) | p=0.59 |
| Alzheimer´s disease cases |  | n=868 |  | n=235 |  | n=209 |  | n=211 |  | n=213 |  |
| HR first 3 y after CVD | 1.37 | (0.92-2.04) | 1.55 | (0.73-3.30) | 1.14 | (0.48-2.74) | 0.98 | (0.36-2.64) | 1.65 | (0.85-3.20) | p=0.15 |
| HR > 3 y after CVD | 0.87 | (0.66-1.16) | 1.03 | (0.59-1.80) | 1.11 | (0.66-1.85) | 0.83 | (0.48-1.44) | 0.59 | (0.33-1.08) | p=0.13 |
| Vascular dementia cases |  | n=312 |  | n=85 |  | n=83 |  | n=86 |  | n=58 |  |
| HR first 3 y after CVD | **3.80** | **(2.53-5.70)** | 2.46 | (0.97-6.20) | **3.28** | **(1.43-7.55)** | **7.95** | **(4.39-14.39)** | 1.90 | (0.58-6.20) | **p<0.000001** |
| HR > 3 y after CVD | 1.40 | (0.96-2.04) | 0.71 | (0.26-1.93) | 1.00 | (0.46-2.19) | **2.47** | **(1.33-4.60)** | 1.52 | (0.73-3.15) | **p=0.02** |

**d) Using a narrowly defined GRS**

|  | Stratified on CAD genetic risk score | | | | | | | | |
| --- | --- | --- | --- | --- | --- | --- | --- | --- | --- |
|  | **1^st^ quartile** | | **2^nd^ quartile** | | **3^rd^ quartile** | | **4^th^ quartile** | | **Trend p-value** |
| All dementia cases |  | n=419 |  | n=376 |  | n=339 |  | n=296 |  |
| HR first 3 y after CVD | 1.40 | (0.92-2.13) | **2.17** | **(1.48-3.18)** | **1.97** | **(1.31-2.96)** | **2.31** | **(1.56-3.42)** | **p<0.000001** |
| HR > 3 y after CVD | 1.06 | (0.79-1.42) | 0.96 | (0.70-1.31) | 1.27 | (0.94-1.71) | 1.05 | (0.75-1.45) | p=0.31 |
| Alzheimer´s disease cases |  | n=245 |  | n=224 |  | n=205 |  | n=194 |  |
| HR first 3 y after CVD | 1.32 | (0.76-2.32) | 1.64 | (0.95-2.80) | 0.98 | (0.48-1.99) | **1.88** | **(1.14-3.10)** | **p=0.01** |
| HR > 3 y after CVD | 0.98 | (0.65-1.48) | 0.84 | (0.55-1.28) | 0.81 | (0.52-1.28) | 0.72 | (0.47-1.12) | p=0.07 |
| Vascular dementia cases |  | n=93 |  | n=87 |  | n=73 |  | n=59 |  |
| HR first 3 y after CVD | 1.16 | (0.46-2.92) | **2.62** | **(1.28-5.32)** | **4.62** | **(2.47-8.65)** | **3.56** | **(1.63-7.76)** | **p<0.000001** |
| HR > 3 y after CVD | 0.67 | (0.33-1.37) | 1.27 | (0.73-2.23) | **1.93** | **(1.08-3.47)** | **1.95** | **(1.07-3.57)** | **p=0.004** |

**e) Adjusting for Apolipoprotein E genotype**

|  | Total sample | | Stratified on CAD genetic risk score | | | | | | | | |
| --- | --- | --- | --- | --- | --- | --- | --- | --- | --- | --- | --- |
|  |  |  | **1^st^ quartile** | | **2^nd^ quartile** | | **3^rd^ quartile** | | **4^th^ quartile** | | **Trend p-value** |
| All dementia cases |  | n=1372 |  | n=379 |  | n=338 |  | n=351 |  | n=304 |  |
| HR first 3 y after CVD | **2.16** | **[1.54,2.53]** | 1.58 | [0.97,2.57] | **1.73** | **[1.03,2.90]** | **2.97** | **[1.97,4.50]** | **1.76** | **[1.03,3.00]** | **p<0.000001** |
| HR > 3 y after CVD | 1.09 | [0.82,1.23] | 1.02 | [0.68,1.53] | 0.88 | [0.59,1.31] | 1.24 | [0.85,1.80] | 0.90 | [0.58,1.38] | p=0.95 |
| Alzheimer´s disease cases |  | n=834 |  | n=231 |  | n=196 |  | n=204 |  | n=203 |  |
| HR first 3 y after CVD | 1.37 | **[1.08,2.15]** | 1.59 | [0.84,3.02] | 1.24 | [0.58,2.64] | 1.67 | [0.82,3.43] | 1.52 | [0.78,2.97] | **p=0.03** |
| HR > 3 y after CVD | 0.87 | [0.63,1.12] | 0.80 | [0.44,1.45] | 0.85 | [0.49,1.47] | 0.76 | [0.43,1.36] | 0.89 | [0.52,1.53] | p=0.29 |
| Vascular dementia cases |  | n=300 |  | n=81 |  | n=80 |  | n=82 |  | n=57 |  |
| HR first 3 y after CVD | **3.80** | **[1.96,4.46]** | 1.59 | [0.61,4.09] | **2.58** | **[1.08,6.17]** | **6.43** | **[3.54,11.70]** | 1.62 | [0.49,5.32] | **p<0.000001** |
| HR > 3 y after CVD | 1.40 | [0.71,1.58] | 0.66 | [0.26,1.67] | 1.23 | [0.62,2.45] | 1.39 | [0.67,2.87] | 1.06 | [0.45,2.51] | p=0.51 |

Abbreviations: CVD, cardiovascular disease; CAD, coronary artery disease; HR, hazard ratio

Number of cases and hazard ratios (95% confidence intervals) of dementia/Alzheimer´s disease in presence of CVD, for the total sample and stratified by quartiles of genetic risk score for CAD. Bold numbers indicate significance. The models are adjusted for age, sex, education, and diabetes during follow up.

**Supplementary Table 3: Lists of genes of significance for Alzheimer´s disease or coronary artery disease, and with a significant overlap between lipids and Alzheimer´s disease or coronary artery disease**

| **Alzheimer´s disease** | **Coronary artery disease** | **Alzheimer´s disease and lipids** | **Coronary artery disease and lipids** |
| --- | --- | --- | --- |
| ABCA7 | ADAMTS7 | APOC1 | ATXN2 |
| APOC1 | AIDA | APOC2 | C19orf52 |
| APOC2 | ALS2CR8 | APOC4 | CARM1 |
| APOC4 | AS3MT | APOE | CELSR2 |
| APOE | ATXN2 | BCAM | LDLR |
| BCAM | B9D2 | BCL3 | LPA |
| BCL3 | BCAS3 | BLOC1S3 | LPAL2 |
| BIN1 | C19orf52 | C1QTNF4 | LPL |
| BLOC1S3 | C1orf58 | CBLC | MYBPHL |
| C1QTNF4 | C2orf68 | CEACAM16 | PSRC1 |
| CBLC | CARM1 | CEACAM19 | SARS |
| CEACAM16 | CDKN2A | CLPTM1 | SLC22A3 |
| CEACAM19 | CDKN2B | GEMIN7 | SMARCA4 |
| CLPTM1 | CELSR2 | HLA-DRB1 | TMED1 |
| CR1 | CFDP1 | NKPD1 | YIPF2 |
| CR1L | COL4A1 | PVR |  |
| EPHA1 | FAM177B | PVRL2 |  |
| EXOC3L2 | FGD5 | RELB |  |
| FBXO46 | FURIN | SFRS16 |  |
| GEMIN7 | GGCX | TOMM40 |  |
| HBEGF | GUCY1A3 | TRAPPC6A |  |
| HLA-DRB1 | ICA1L | ZNF296 |  |
| MARK4 | LDLR |  |  |
| MS4A2 | LPA |  |  |
| MS4A3 | LPAL2 |  |  |
| MS4A4A | LPL |  |  |
| MS4A6A | MAPK7 |  |  |
| MS4A6E | MAT2A |  |  |
| NKPD1 | MFAP4 |  |  |
| PICALM | MIA3 |  |  |
| PLAC1L | MORF4L1 |  |  |
| PVR | MRAS |  |  |
| PVRL2 | MYBPHL |  |  |
| QPCTL | PHACTR1 |  |  |
| RELB | PLG |  |  |
| SFRS16 | PPAP2B |  |  |
| SLC4A9 | PSRC1 |  |  |
| TAS2R60 | RNF112 |  |  |
| TOMM40 | RNF181 |  |  |
| TRAPPC6A | SARS |  |  |
| ZNF296 | SLC22A2 |  |  |
| ZYX | SLC22A3 |  |  |
|  | SMARCA4 |  |  |
|  | SMG6 |  |  |
|  | TAF1A |  |  |
|  | TCF21 |  |  |
|  | TGFB1 |  |  |
|  | TMED1 |  |  |
|  | TMEM150 |  |  |
|  | TMEM170A |  |  |
|  | VAMP5 |  |  |
|  | VAMP8 |  |  |
|  | WDR12 |  |  |
|  | YIPF2 |  |  |

**Supplementary Table 4: Pathway analyses**

1. **Pathways enriched for genes involved in Alzheimer´s disease and lipid fractions**

| **p-value** | **q-value** | **Pathway** | **source** | **members_input_ overlap** | **members_input_ overlap_geneids** | **size** | **Effective _size** |
| --- | --- | --- | --- | --- | --- | --- | --- |
| 2.14E-06 | 5.82E-05 | Statin Pathway, Pharmacodynamics* | PharmGKB | APOE; APOC2; APOC1 | 341; 344; 348 | 25 | 25 |
| 4.16E-06 | 5.82E-05 | Statin Pathway* | Wikipathways | APOE; APOC2; APOC1 | 344; 341; 348 | 31 | 31 |
| 2.21E-05 | 2.06E-04 | Nectin/Necl trans heterodimerization | Reactome | PVR; PVRL2 | 5819; 5817 | 7 | 7 |
| 1.10E-04 | 7.68E-04 | HDL-mediated lipid transport | Reactome | APOE; APOC2 | 344; 348 | 15 | 15 |
| 1.42E-04 | 7.95E-04 | Chylomicron-mediated lipid transport | Reactome | APOE; APOC2 | 344; 348 | 17 | 17 |
| 4.04E-04 | 1.40E-03 | Cell adhesion molecules (CAMs) - Homo sapiens (human) | KEGG | PVR; HLA-DRB1; PVRL2 | 3123; 5817; 5819 | 142 | 142 |
| 4.51E-04 | 1.40E-03 | Lipoprotein metabolism | Reactome | APOE; APOC2 | 344; 348 | 30 | 30 |
| 4.51E-04 | 1.40E-03 | Adherens junctions interactions | Reactome | PVR; PVRL2 | 5819; 5817 | 30 | 30 |
| 4.51E-04 | 1.40E-03 | Nectin adhesion pathway | PID | PVR; PVRL2 | 5819; 5817 | 30 | 30 |
| 8.85E-04 | 2.48E-03 | Retinoid metabolism and transport | Reactome | APOE; APOC2 | 344; 348 | 42 | 42 |
| 1.41E-03 | 3.58E-03 | Apoptosis-related network due to altered Notch3 in ovarian cancer | Wikipathways | APOE; BCL3 | 602; 348 | 53 | 53 |
| 1.68E-03 | 3.88E-03 | Lipid digestion, mobilization, and transport | Reactome | APOE; APOC2 | 344; 348 | 58 | 58 |
| 1.80E-03 | 3.88E-03 | Cell-cell junction organization | Reactome | PVR; PVRL2 | 5819; 5817 | 60 | 60 |
| 2.05E-03 | 4.09E-03 | IL12-mediated signaling events | PID | RELB; HLA-DRB1 | 5971; 3123 | 65 | 64 |
| 3.66E-03 | 6.83E-03 | Cell junction organization | Reactome | PVR; PVRL2 | 5817; 5819 | 86 | 86 |
| 4.54E-03 | 7.95E-03 | Visual phototransduction | Reactome | APOE; APOC2 | 348; 344 | 96 | 96 |
| 7.94E-03 | 1.31E-02 | Cell-Cell communication | Reactome | PVR; PVRL2 | 5817; 5819 | 128 | 128 |
| 9.83E-03 | 1.53E-02 | Immunoregulatory interactions between a Lymphoid and a non-Lymphoid cell | Reactome | PVR; PVRL2 | 5817; 5819 | 146 | 143 |

1. **Pathways enriched for genes involved in coronary artery disease and lipid fractions**

| **p-value** | **q-value** | **Pathway** | **Source** | **members_input_overlap** | **members_input_overlap_geneids** | **size** | **effective_size** |
| --- | --- | --- | --- | --- | --- | --- | --- |
| 2.06E-06 | 3.70E-05 | Lipoprotein metabolism | Reactome | LDLR; LPL; LPA | 4018; 3949; 4023 | 30 | 30 |
| 1.08E-05 | 9.26E-05 | LDL-mediated lipid transport | Reactome | LDLR; LPA | 3949; 4018 | 6 | 6 |
| 1.54E-05 | 9.26E-05 | Lipid digestion, mobilization, and transport | Reactome | LDLR; LPL; LPA | 4018; 3949; 4023 | 58 | 58 |
| 9.70E-05 | 4.36E-04 | Chylomicron-mediated lipid transport | Reactome | LDLR; LPL | 3949; 4023 | 17 | 17 |
| 2.13E-04 | 7.67E-04 | Statin Pathway, Pharmacodynamics* | PharmGKB | LDLR; LPL | 4023; 3949 | 25 | 25 |
| 3.29E-04 | 9.88E-04 | Statin Pathway* | Wikipathways | LDLR; LPL | 3949; 4023 | 31 | 31 |
| 6.07E-04 | 1.50E-03 | Retinoid metabolism and transport | Reactome | LDLR; LPL | 3949; 4023 | 42 | 42 |
| 7.28E-04 | 1.50E-03 | Aryl Hydrocarbon Receptor | Wikipathways | PSRC1; LPL | 4023; 84722 | 46 | 46 |
| 7.50E-04 | 1.50E-03 | Metabolism of lipids and lipoproteins | Reactome | LDLR; LPL; CARM1; LPA | 4018; 4023; 3949; 10498 | 517 | 516 |
| 1.59E-03 | 2.85E-03 | SREBP signalling | Wikipathways | LDLR; LPL | 3949; 4023 | 68 | 68 |
| 1.78E-03 | 2.90E-03 | RMTs methylate histone arginines | Reactome | SMARCA4; CARM1 | 6597; 10498 | 72 | 72 |
| 3.13E-03 | 4.70E-03 | Visual phototransduction | Reactome | LDLR; LPL | 4023; 3949 | 96 | 96 |
| 5.58E-03 | 7.72E-03 | Metabolism | Reactome | LDLR; LPL; CARM1; LPA; SLC22A3 | 4018; 6581; 4023; 3949; 10498 | 1481 | 1474 |
| 6.63E-03 | 8.53E-03 | Direct p53 effectors | PID | SMARCA4; CARM1 | 6597; 10498 | 141 | 141 |

Results from pathway analyses of genes presented in table S3 significant for both lipids and Alzheimer´s disease (a) or coronary artery disease (b).

***** Statin Pathway, Pharmacodynamics and Statin Pathway are considered as the same pathway

**Supplementary Table 5: SNPs used in the genetic risk score for coronary artery disease**

| **Locus name** | **SNP** | **Effect allele (frequency)** | **CardioMetabochip** | **r2** |
| --- | --- | --- | --- | --- |
| PCSK9 | rs11206510 | T (0.85) |  |  |
| PPAP2B | rs17114036 | A (0.92) | rs9970807 | 0.90 |
| SORT1 | rs646776 | T (0.75) |  |  |
| IL6R | rs4845625 | T (0.45) |  |  |
| MIA3 | rs17465637 | C (0.66) |  |  |
| AK097927 | rs16986953 | A (0.10) |  |  |
| APOB | rs515135 | C (0.79) |  |  |
| ABCG5-ABCG8 | rs6544713 | T (0.32) |  |  |
| VAMP5-VAMP8-GGCX | rs1561198 T | T (0.46) |  |  |
| ZEB2-ACO74093.1 | rs2252641 C | C (0.48) |  |  |
| WDR12 | rs6725887 | C (0.11) |  |  |
| MRAS | rs9818870 | T (0.14) |  |  |
| EDNRA | rs1878406 | T (0.16) |  |  |
| GUCY1A3 | rs7692387 | G (0.81) |  |  |
| SLC22A4-SLC22A5 | rs273909 | G (0.12) |  |  |
| PHACTR1 | rs12526453 | C (0.71) | rs9369640 | 0.90 |
| ANKS1A | rs17609940 | G (0.82) | rs2077750 | 1.00 |
| KCNK5 | rs10947789 | T (0.78) |  |  |
| TCF21* | rs12190287 | C (0.62) | Value imputed |  |
| SLC22A3-LPAL2-LPA | rs2048327 | C (0.35) |  |  |
| PLG | rs4252120 | T (0.74) |  |  |
| HDAC9 | rs2023938 | C (0.10) |  |  |
| 7q22 | rs10953541 | C (0.78) | Value imputed |  |
| ZC3HC1 | rs11556924 | C (0.69) |  |  |
| LPL | rs264 | G (0.85) |  |  |
| TRIB1 | rs2954029 | A (0.55) |  |  |
| 9p21 | rs4977574 | G (0.49) | rs1537371 | 1.00 |
| ABO | rs579459 | C (0.21) |  |  |
| KIAA1462 | rs2505083 | C (0.40) |  |  |
| CXCL12 | rs501120 | T (0.81) | rs671765 | 1.00 |
| LIPA | rs1412444 | T (0.37) | rs2246833 | 1.00 |
| CYP17A1-CNNM2-NT5C2 | rs12413409 | G (0.89) |  |  |
| PDGFD | rs974819 | T (0.33) |  |  |
| ZNF259-APOA5-APOA1 | rs964184 | G (0.18) | Value imputed |  |
| ATP2B1 | rs7136259 | T (0.43) |  |  |
| SH2B3 | rs3184504 | T (0.42) |  |  |
| FLT1 | rs9319428 | A (0.31) |  |  |
| COL4A1/A2 | rs9515203 | T (0.76) |  |  |
| HHIPL1 | rs2895811 | C (0.41) |  |  |
| ADAMTS7 | rs7173743 | T (0.56) |  |  |
| FURIN-FES | rs17514846 | A (0.44) |  |  |
| SMG6 | rs216172 | C (0.35) | rs143499 | 1.00 |
| RAI1-PEMT-RASD1 | rs12936587 | G (0.61) |  |  |
| UBE2Z | rs46522 | T (0.51) | rs15563 | 0.94 |
| LDLR | rs1122608 | G (0.77) |  |  |
| APOE-APOC1 | rs2075650 | G (0.13) |  |  |
| KCNE2 (gene desert) | rs9982601 | T (0.13) |  |  |
| REST-NOA1 | rs17087335 | T (0.21) |  |  |
| NOS3 | rs3918226 | T (0.06) | Value imputed |  |
| SWAP70 | rs10840293 | A (0.55) | rs93139 | 0.97 |
| SMAD3 | rs56062135 | C (0.79) | rs16950687 | 0.95 |
| MFGE8-ABHD2 | rs8042271 | G (0.9) | Value imputed |  |
| BCAS3 | rs7212798 | C (0.15) | Value imputed |  |
| PMAIP1-MC4R | rs663129 | A (0.26) | rs571312 | 0.91 |
| POM121L9P-ADORA2A | rs180803 | G (0.97) | Value imputed |  |

List of the 55 SNPs used in the genetic risk score for coronary artery disease, with effect allele and effect allele frequency. The column for CardioMetabochip indicates SNPs not present on the chip, and whether a proxy was used or if the value was imputed.

**Supplementary Figure 1: Scatter plot of genetic correlations between Alzheimer's disease (horizontal axis) and coronary artery disease (vertical axis) with their shared risk factors: BMI, T2D, and the lipid fractions LDL, HDL, triglycerides, and total cholesterol. Based on results from Bulik-Sullivan et al. 2015^21^.**
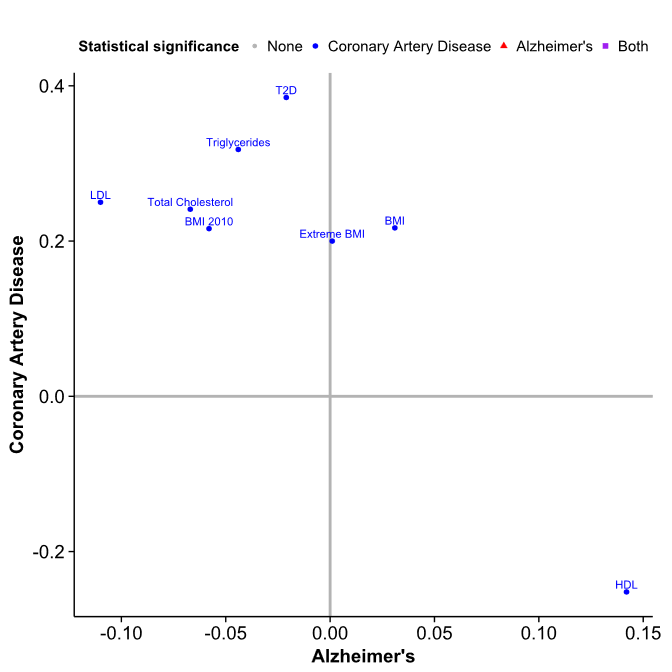


Abbreviations: AD, Alzheimer´s disease; CAD, coronary artery disease; T2D, type 2 diabetes; BMI, body mass index; LDL, low density lipoprotein; HDL, high density lipoprotein

Genetic correlations between AD and risk factors are shown on the x-axis, and genetic correlations between CAD and risk factors on the y-axis. Values above zero imply a positive correlation and values below zero a negative correlation. Label colors indicate statistical significance at 5% level. Please note that significant correlations were only found for CAD.

**References**

1. Socialstyrelsen. Värdering av diagnoskvaliteten för akut hjärtinfarkt i patientregistret 1987 och 1995. [*http://wwwsocialstyrelsense/Lists/Artikelkatalog/Attachments/17891/2000-0-100pdf*](http://wwwsocialstyrelsense/Lists/Artikelkatalog/Attachments/17891/2000-0-100pdf) 2000.

2. Gatz M, Fratiglioni L, Johansson B, Berg S, Mortimer JA, Reynolds CA *et al.* Complete ascertainment of dementia in the Swedish Twin Registry: the HARMONY study. *Neurobiol Aging* 2005; **26**(4)**:** 439-447.

3. Folstein MF, Folstein SE, McHugh PR. "Mini-mental state". A practical method for grading the cognitive state of patients for the clinician. *J Psychiatr Res* 1975; **12**(3)**:** 189-198.

4. Gatz M, Reynolds CA, John R, Johansson B, Mortimer JA, Pedersen NL. Telephone screening to identify potential dementia cases in a population-based sample of older adults. *Int Psychogeriatr* 2002; **14**(3)**:** 273-289.

5. American Psychiatric Association. *Diagnostic and statistical manual of mental disorders : DSM-III-R*. American Psychiatric Association1987.

6. American Psychiatric Association. *Diagnostic and statistical manual of mental disorders : DSM-IV*. American Psychiatric Association1994.

7. McKhann G, Drachman D, Folstein M, Katzman R, Price D, Stadlan EM. Clinical diagnosis of Alzheimer's disease: report of the NINCDS-ADRDA Work Group under the auspices of Department of Health and Human Services Task Force on Alzheimer's Disease. *Neurology* 1984; **34**(7)**:** 939-944.

8. Roman GC, Tatemichi TK, Erkinjuntti T, Cummings JL, Masdeu JC, Garcia JH *et al.* Vascular dementia: diagnostic criteria for research studies. Report of the NINDS-AIREN International Workshop. *Neurology* 1993; **43**(2)**:** 250-260.

9. 1000 Genomes Project Consortium, Abecasis GR, Auton A, Brooks LD, DePristo MA, Durbin RM *et al.* An integrated map of genetic variation from 1,092 human genomes. *Nature* 2012; **491**(7422)**:** 56-65.

10. Voight BF, Kang HM, Ding J, Palmer CD, Sidore C, Chines PS *et al.* The metabochip, a custom genotyping array for genetic studies of metabolic, cardiovascular, and anthropometric traits. *PLoS Genet* 2012; **8**(8)**:** e1002793.

11. Radmanesh F, Devan WJ, Anderson CD, Rosand J, Falcone GJ, Alzheimer's Disease Neuroimaging I. Accuracy of imputation to infer unobserved APOE epsilon alleles in genome-wide genotyping data. *Eur J Hum Genet* 2014; **22**(10)**:** 1239-1242.

12. Lambert JC, Ibrahim-Verbaas CA, Harold D, Naj AC, Sims R, Bellenguez C *et al.* Meta-analysis of 74,046 individuals identifies 11 new susceptibility loci for Alzheimer's disease. *Nat Genet* 2013; **45**(12)**:** 1452-1458.

13. Nikpay M, Goel A, Won HH, Hall LM, Willenborg C, Kanoni S *et al.* A comprehensive 1,000 Genomes-based genome-wide association meta-analysis of coronary artery disease. *Nat Genet* 2015; **47**(10)**:** 1121-1130.

14. Locke AE, Kahali B, Berndt SI, Justice AE, Pers TH, Day FR *et al.* Genetic studies of body mass index yield new insights for obesity biology. *Nature* 2015; **518**(7538)**:** 197-206.

15. Morris AP, Voight BF, Teslovich TM, Ferreira T, Segre AV, Steinthorsdottir V *et al.* Large-scale association analysis provides insights into the genetic architecture and pathophysiology of type 2 diabetes. *Nat Genet* 2012; **44**(9)**:** 981-990.

16. International Consortium for Blood Pressure Genome-Wide Association S, Ehret GB, Munroe PB, Rice KM, Bochud M, Johnson AD *et al.* Genetic variants in novel pathways influence blood pressure and cardiovascular disease risk. *Nature* 2011; **478**(7367)**:** 103-109.

17. Teslovich TM, Musunuru K, Smith AV, Edmondson AC, Stylianou IM, Koseki M *et al.* Biological, clinical and population relevance of 95 loci for blood lipids. *Nature* 2010; **466**(7307)**:** 707-713.

18. Liu JZ, McRae AF, Nyholt DR, Medland SE, Wray NR, Brown KM *et al.* A versatile gene-based test for genome-wide association studies. *Am J Hum Genet* 2010; **87**(1)**:** 139-145.

19. Ploner A. VEGAStools: Basic manipulations of VEGAS gene lists. . *R package version 0009000* [*https://githubcom/alexploner/VEGAStools*](https://githubcom/alexploner/VEGAStools).

20. Kamburov A, Stelzl U, Lehrach H, Herwig R. The ConsensusPathDB interaction database: 2013 update. *Nucleic Acids Res* 2013; **41**(Database issue)**:** D793-800.

21. Bulik-Sullivan B, Finucane HK, Anttila V, Gusev A, Day FR, Loh PR *et al.* An atlas of genetic correlations across human diseases and traits. *Nat Genet* 2015; **47**(11)**:** 1236-1241.
